# Late Occurring Ectopic Pregnancy in a Posthysterectomy Patient

**DOI:** 10.1155/2013/975196

**Published:** 2013-09-18

**Authors:** Munazza Anis, Abid Irshad, Susan Ackerman

**Affiliations:** Medical University of South Carolina, Department of Radiology, Charleston, SC 29425, USA

## Abstract

The incidence of ectopic pregnancy after hysterectomy is extremely rare with only 56 cases reported in the medical literature. Due to its rare occurrence, this diagnosis may not be initially considered when such a patient presents with abdominopelvic pain. It is an important diagnosis to keep in mind since a delay in diagnosis may lead to death. The case presented below describes this extremely unusual diagnosis of an ectopic pregnancy which occurred six years after a supracervical hysterectomy.

## 1. Introduction

Posthysterectomy ectopic pregnancy cases can be classified as early (preexisting pregnancy) or late occurring based on the presence or absence of an unrecognized pregnancy at the time of hysterectomy [1]. Among the 56 reported cases of posthysterectomy ectopic pregnancies in the literature, less than half occurred in late posthysterectomy period. Pregnancy can occur after virtually any type of hysterectomy, and the patients may present with acute or subacute symptoms with or without vaginal bleeding.

## 2. Case Report

A 36-year-old Latin American woman, G3 P3, had initially presented to the emergency department with a two-week history of bilateral lower quadrant pain at an outside hospital from which she was referred to out institution after initial testing. The pain had started 2 weeks ago coinciding with mild vaginal bleeding. She denied any other symptomatology. She had undergone a cesarean section for fetal distress during her third pregnancy (G3) which was followed by cesarean hysterectomy due to uncontrollable bleeding. A supracervical hysterectomy was performed at that time which was approximately 6 years ago. Physical examination revealed normal vital signs. Her abdomen was soft and mildly tender to palpation. Recent laboratory studies from an outside lab showed a beta-HCG level of 10,587 mIU/mL (normal: 0–4). Initially, an ultrasound was obtained which was followed by an MRI examination for further characterization.

**Figure 1:**
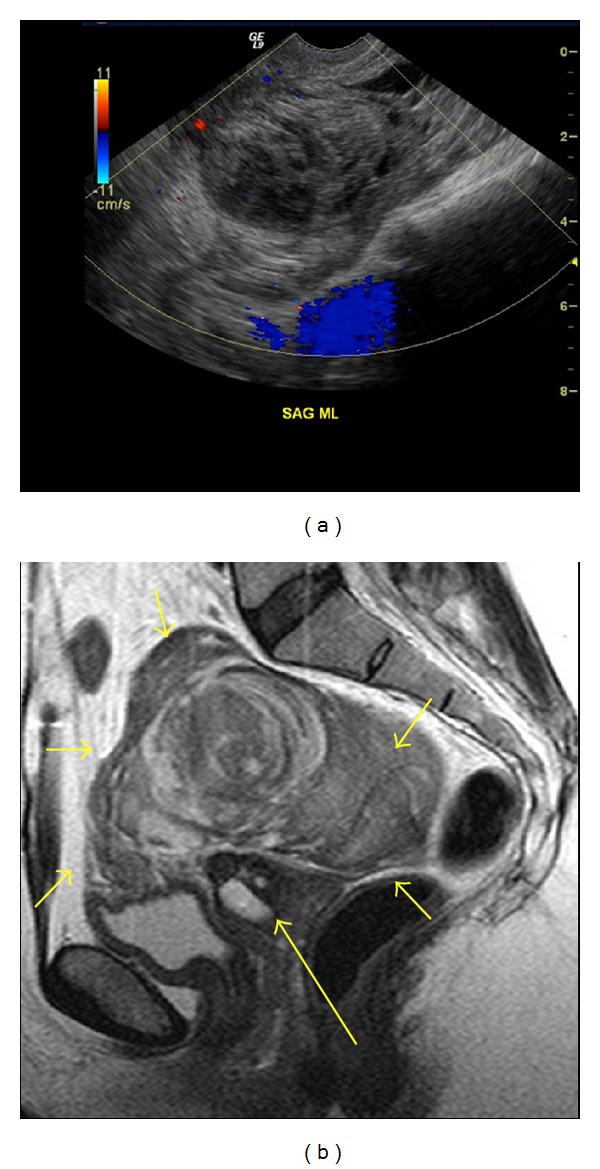
Image (a) is a transvaginal sagittal image with Doppler through the midline pelvis demonstrating a large central heterogeneous mass in the pelvis which does not show internal flow likely representing an organizing hematoma. Image (b) is a T2 weighted sagittal MR image through pelvis showing a large central pelvic hematoma (small arrows). A prominent cervical stump containing a nabothian cyst (large arrow) is noted posterior to the bladder.

**Figure 2:**
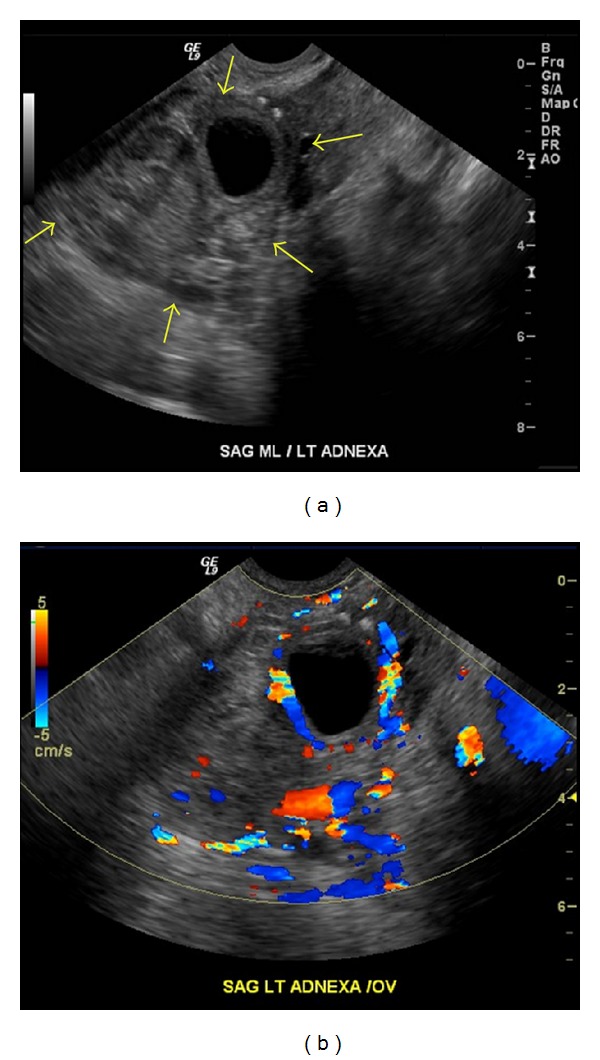
Image (a) is a transvaginal sagittal US image through the left adnexal region which demonstrates a heterogeneous mass (arrows) containing an anechoic cystic structure with slightly echogenic thick walls. Image (b) is a Doppler US image through the same area demonstrating an intense peripheral vascularity with a “ring of fire” appearance.

On transabdominal ultrasound, uterus was absent. A 10 cm heterogeneous mass was present in the midline pelvis without any significant Doppler flow. Additionally, there was a 5 cm complex heterogeneous left adnexal mass without any Doppler flow. Transvaginal ultrasound also demonstrated the large heterogeneous mass measuring 10 cm × 6.5 cm × 8.4 cm in the central pelvis with no color flow on Doppler examination later confirmed with MRI (Figures 1(a) and 1(b)). It was thought to represent an organized hematoma and less likely a neoplasm. Additionally, the left adnexal region demonstrated a 5.2 cm × 3.8 cm × 4.1 cm heterogeneous mass containing an anechoic, well-circumscribed, rounded, cystic structure measuring 2.3 cm × 2.5 cm × 2.8 cm (Figure 2(a)). The anechoic cystic structure showed slightly thick and echogenic walls with an encircling vascularity on color Doppler exam (an appearance frequently termed as “ring of fire” appearance) (Figure 2(b)). This was reported to either represent a corpus luteum cyst of the left ovary or an ectopic gestational sac. A normal ovary was not clearly visualized either by transabdominal or transvaginal approach on either sides. 

**Figure 3:**
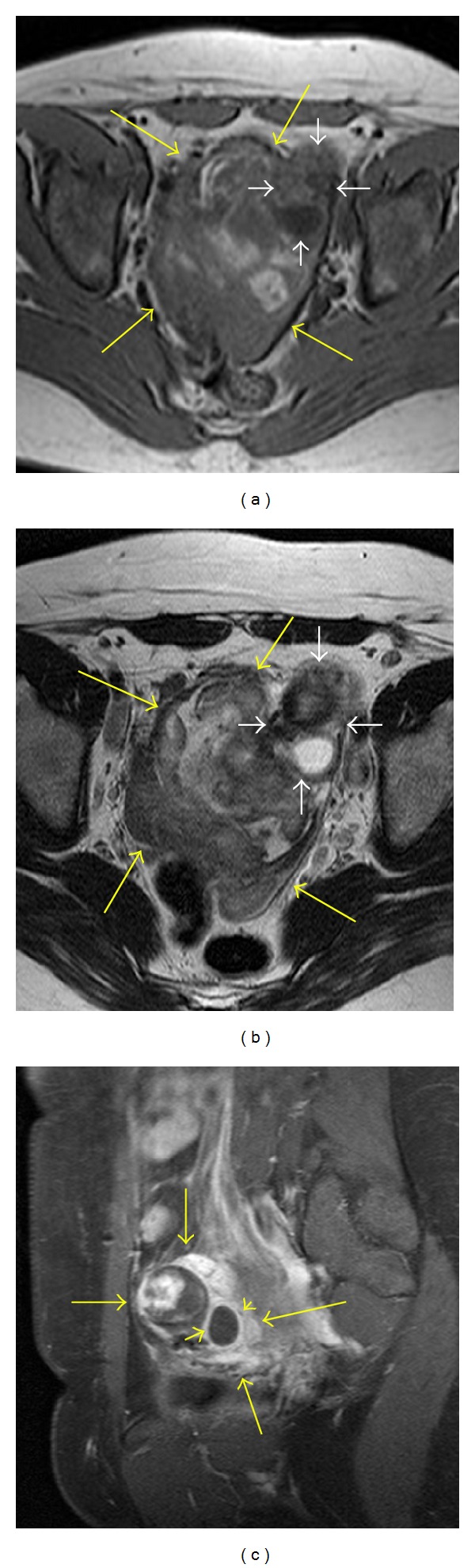
Image (a) is a noncontrast T1 axial image through the pelvis which demonstrates a large pelvic hematoma (large arrows) showing heterogeneous hyperintense signal related to the blood products. An additional smaller tubo-ovarian mass is noted in the left pelvis (small white arrows) which contains a posterior cystic component. Image (b) is a T2 axial image through the same area showing central hematoma (large arrows) and left tubo-ovarian mass (small arrows) with cystic component. Image (c) is a postgadolinium sagittal image through the left adnexa which shows heterogeneous enhancement of the tubo-ovarian complex mass (large arrows). The posterior aspect of this mass shows a thick-walled cystic structure with enhancement of the wall (small arrows). This corresponds to the cystic structure seen on US with “ring of fire” appearance in Figure 2(b).

MRI depicted postsurgical changes from supracervical hysterectomy with a nonenhancing T1 hyperintense and T2 hypointense mass in the midpelvis measuring approximately 11 cm × 10 cm compatible with blood products (Figures 3(a) and 3(b)). Additionally, there was a left pelvic mass which showed heterogeneous enhancement and was considered to represent a tubo-ovarian mass complex (Figures 3(a), 3(b) and 3(c)). There was a rounded thick-walled cystic structure along the anterior aspect which showed wall enhancement (Figure 3(c)). 

Patient was taken to the operating room for laparoscopic surgery. Hemoperitoneum was evacuated, and omental adhesions to the anterior abdominal wall were dissected free. Multiple adhesions were noted in the left pelvis, with enlarged left fallopian tube and ovary surrounded by clots. The right adnexa was not visualized and appeared to be surgically absent. The left adnexa was adhered to the vaginal cuff. The adhesions were dissected, the pedicle was excised, and the specimen of the tubo-ovarian complex was obtained and sent to pathology. Patient had an uncomplicated postoperative course. She was discharged from the hospital on postoperative day 1 with appropriate instructions. At discharge, her beta-HCG was noted to have decreased to 4401 mIU/mL from 10,587 mIU/mL. 

On pathologic examination of surgical specimens, midpelvic tissue sample was identified to be fat and organized blood. Left ovary showed chorionic villi and blood. The diagnosis was made of a tubo-ovarian complex with ectopic pregnancy within the left ovary.

On post-operative follow-up visit one month later, a repeat measurement of Quantitative beta-HCG showed a normal level of <3.0 mIU/mL (normal: 0–4).

## 3. Discussion

Preexisting or early presenting ectopic pregnancy after hysterectomy can occur after virtually every kind of hysterectomy [1]. Theories postulate a prefertilized ovum in the fallopian tube spilling in the peritoneal cavity during hysterectomy [1–3]. Symptoms of ectopic pregnancy closely mimic the symptoms of common post hysterectomy complications such as pelvic hematoma or vaginal cuff infection. As a result, ectopic pregnancy is rarely suspected until the diagnosis is made by additional tests or repeat surgery [4–6]. One way to prevent early posthysterectomy ectopic pregnancy is to take measures to prevent pregnancy before hysterectomy. Hysterectomy should not be performed in the luteal phase of the menstrual cycle unless the patient is previously sterilized, using reliable contraception, or abstaining from vaginal intercourse in the preoperative period. 

 There have been 25 reported cases of late-presentation ectopic pregnancies after hysterectomy occurring as late as 12 years after hysterectomy. It is believed that late-presenting posthysterectomy ectopic pregnancy occurs when sperm gains access to an ovulated ovum through a fistulous tract between the vagina and the peritoneal cavity. This tract can often be diagnosed by fistulography [7, 8] or MRI examination. It is thought that an open vaginal cuff closure technique, vaginal cuff infection, hematoma formation after hysterectomy, vaginal cuff granulation tissue, and a prolapsed fallopian tube increase the chance of vaginal-to-peritoneal fistula formation [8–11]. The technique used to close the vaginal cuff during vaginal hysterectomy brings the adnexal structures in closer proximity to the vaginal cuff as compared to the method used to close the vaginal cuff during abdominal hysterectomy [8, 10]. This difference in technique could potentially contribute to the development of a fistula. 

In subtotal hysterectomy, chances of a fistulous tract formation may be increased by leaving a remnant of cervix (as in our case) or the epithelialization of a much larger vaginal cuff closure area due to cervical dilation at the time of cesarean hysterectomy [1, 10]. It is believed that the incidence of ectopic pregnancy could potentially be increased by laparoscopic hysterectomy which is now increasingly performed [1, 11]. In this type of hysterectomy, the residual proximal cervical canal is cauterized to prevent cyclic vaginal bleeding which may not be adequate to prevent patency of the cervical canal.

## 4. Conclusion

In conclusion, it is imperative to maintain a high index of suspicion for pregnancy in a posthysterectomy patient presenting with acute abdominal pain if the ovaries are in situ. MRI can help not only in making the diagnosis of an ectopic after hysterectomy but also in diagnosing a vaginal cuff fistula, adhered adnexal structures to the vaginal cuff, and in diagnosing a prominent cervical remnant.
